# TB-HIV co-infection: spatial and temporal distribution in the largest Brazilian metropolis

**DOI:** 10.11606/s1518-8787.2020054002108

**Published:** 2020-10-28

**Authors:** Roberta Figueiredo Cavalin, Alessandra Cristina Guedes Pellini, Regina Rocha Gomes de Lemos, Ana Paula Sayuri Sato

**Affiliations:** I Universidade de São Paulo Faculdade de Saúde Pública Programa de Pós-Graduação em Saúde Pública São PauloSP Brasil Universidade de São Paulo . Faculdade de Saúde Pública . Programa de Pós-Graduação em Saúde Pública . São Paulo , SP , Brasil; II Universidade Nove de Julho Faculdade de Medicina Diretoria de Ciências Médicas São PauloSP Brasil Universidade Nove de Julho . Faculdade de Medicina . Diretoria de Ciências Médicas . São Paulo , SP , Brasil; III Secretaria Municipal da Saúde Coordenação de Vigilância em Saúde de São Paulo Programa Municipal de Controle da Tuberculose São PauloSP Brasil Secretaria Municipal da Saúde . Coordenação de Vigilância em Saúde de São Paulo . Programa Municipal de Controle da Tuberculose . São Paulo , SP , Brasil; IV Universidade de São Paulo Faculdade de Saúde Pública Departamento de Epidemiologia São PauloSP Brasil Universidade de São Paulo . Faculdade de Saúde Pública . Departamento de Epidemiologia . São Paulo , SP , Brasil

**Keywords:** Tuberculosis, HIV infections, epidemiology, Spatial analysis, Time series studies

## Abstract

**OBJECTIVE:**

To describe the spatial and temporal distribution of TB-HIV co-infection, as well as the profile of the characteristics of the co-infected population in the municipality of São Paulo.

**METHODS:**

This is an ecological and time series study with data from the Tuberculosis Patient Control System (TBWeb), including all new cases of tuberculosis co-infected individuals with HIV living in the municipality from 2007 to 2015. Time trends of the disease were analyzed using Prais-Winsten regression. The cases were geocoded by the address of residence for the elaboration of maps with the incidence rates smoothed by the local empirical Bayesian method. The global and local Moran indexes evaluated spatial autocorrelation. Individuals’ profiles were described and the characteristics of the cases with and without fixed residence were compared by Pearson’s chi-square or Fisher’s exact tests.

**RESULTS:**

We analyzed 6,092 new cases of TB-HIV co-infection (5,609 with fixed residence and 483 without fixed residence). The proportion of TB-HIV co-infection ranged from 10.5% to 13.7%, with a drop of 3.0% per year (95%CI -3.4 – -2.6) and was higher in individuals without fixed residence. Incidence rates decreased by 3.6% per year (95%CI -4.4% – -2.7%), declining from 7.0 to 5.3 per 100,000 inhabitants/year. Co-infection showed positive and significant spatial autocorrelation, with heterogeneous spatial pattern and a high-risk cluster in the central region of the municipality. Cure was achieved in 55.5% of cases with fixed residence and in 32.7% of those without a fixed residence.

**CONCLUSIONS:**

The data indicate an important advance in the control of TB-HIV co-infection in the period analyzed. However, we identified areas and populations that were unequally affected by the disease and that should be prioritized in the improvement of actions to prevent and control co-infection.

## INTRODUCTION

Despite being the oldest infectious diseases in human history, tuberculosis (TB) still poses a major challenge to global public health, which affected 10.0 million people and caused 1.4 million deaths in 2018 ^1^ . Followed by acquired immunodeficiency syndrome (AIDS), TB is the most important cause of mortality by infectious agent in the world ^1^ .

Beginning in the 1980s, the AIDS epidemic strongly affected the epidemiological profile and TB control. The immunological impairment caused by HIV/AIDS favors the multiplication *of M. tuberculosis* and the illness of TB ^2^ , and HIV infection is the most important risk factor for active TB, with a 19-fold higher risk in the virus-infected population compared to the general population ^1^ . The synergy between diseases is also observed in HIV-related mortality, with TB being the leading cause of death among people living with HIV/AIDS ^3^ .

In 2018, about 8.6% of all TB cases in the world were HIV-positive, totaling 862,000 people affected by co-infection ^1^ . TB-HIV co-infection strongly afflicts people living in precarious living conditions, who suffer from the lack of resources for the prevention, diagnosis, treatment and control of both TB and HIV/AIDS ^1 , 4^ . Co-infection especially affects underdeveloped and populated regions, such as some areas of the African continent, where it represents more than 50% of cases ^1^ .

In 2017, Brazil had 11.4% of new TB cases co-infected with the HIV virus, and the state of São Paulo identified 9.3% of co-infection ^5^ . Considering the classification of the World Health Organization ^1^ , which defines the countries with the highest TB burden, the current epidemiological situation in Brazil includes the country in the contexts of high burden of TB and the disease associated with HIV, being, therefore, one of the priority countries for investments in control actions.

Despite the relevance of co-infection, there are still few publications on its spatial and temporal distribution in Brazil ^6^ . Geographic information systems (GIS) are valuable tools for the analysis of spatial data in the health area, and their use can contribute to epidemiological surveillance of transmissible diseases such as TB and HIV/AIDS, since they facilitate knowledge of the distribution of cases in the territory and allow investigating the factors associated with transmission and identifying priority areas for interventions ^10^ . In health, time measurement is also very useful for understanding the study object. Time series, which are ways of organizing quantitative information in time ^11^ , can be used to characterize the time trends of injuries that influence populations’ health, as well as to evaluate the effectiveness of control policies.

Revealing intense social inequalities ^12^ , with a complex network of health care and the highest demographic density in the country ^13^ , the municipality of São Paulo (MSP) has the highest number of cases of TB-HIV co-infection in Brazil ^5^ and, therefore, demands knowledge of the dynamics of the disease to improve control actions. In this sense, our study describes the spatial and temporal distribution of TB-HIV co-infection, as well as the profile of the characteristics of the co-infected population in the MSP.

## METHODS

### Study Design and Area

This is an ecological study with analytical component and time series, developed in the MSP, located in the Southeast region of Brazil, which is the most populous municipality in the country, with a total of 11,638,802 inhabitants in 2016 and almost all of the population residing in the urban area (99.1%) ^14^ . TB control actions is conducted by the *Programa Municipal de Controle da Tuberculose* (PMCT – Municipal Tuberculosis Control Program), in a decentralized manner ^15^ .

### Population and Study Period

We included all new cases of TB co-infected with the HIV virus residing in the MSP and with incidence in the period from 2007 to 2015, except those with diagnostic changes and patients deprived of liberty. The individual that provided a fixed housing address at the time of TB notification was defined as “case with fixed residence”, and the “case without fixed residence” was defined as the individual with no fixed housing address in the TB notification form (FN-TB). Our study analyzed separately the populations with and without fixed residence, considering that the singular vulnerabilities that integrate the lives of homeless individuals can influence the health-disease process, especially in TB, which is a socially determined disease ^16^ .

### Data Sources

Data from TB-HIV co-infection cases were extracted on June 5, 2017 from the Tuberculosis Patient Control System (TBWeb) of the São Paulo State Department of Health, which stores FN-TB information. The digital base of street workers and the digital map of the administrative districts (AD) of the MSP were obtained from the *Centro de Estudos da Metrópole* (CEM – Center for Metropolitan Studies) and the Coordination of Epidemiology and Information of the Municipal Health Department of São Paulo (CEInfo). Data on the resident population of the MSP of the *Fundação Sistema Estadual de Análise de Dados* (Seade – State Data Analysis System Foundation) were also used.

### Analyses

The proportion of TB-HIV co-infection among new TB cases was estimated for each year of the study, using the total number of new TB cases and the number of cases with HIV-positive serology, according to TBWeb. To estimate the annual incidence rate, we considered the total number of new cases of TB-HIV co-infection per year, divided by the total resident population estimated in the middle of the same year, multiplied by 100,000, resulting in an incidence rate per 100,000 inhabitants/year.

To verify the temporal trend of the proportions and incidence rates of TB-HIV co-infection in the municipality, we constructed generalized linear regression models using the Prais-Winsten method ^11^ . To quantitatively estimate the time trends of co-infection in the analyzed period, we used the formula of annual percent change (APC), as well as a 95% confidence interval (95%CI) ^11^ . The linear regression models were constructed in Stata software, version 12.

To characterize the spatial distribution patterns of TB-HIV co-infection, individuals were georeferenced based on their home address. The spatial unit of choice was the Administrative District (AD), which is the smallest administrative division of the MSP. For all spatial analyses, the data were presented according to the three-year incidence of the disease (2007–2009, 2010–2012 and 2013–2015), thus incorporating the temporal approach to verify spatial changes that occurred during the study period and the patterns of diffusion of the disease. The division of the *Coordenadorias Regionais de Saúde* (CRS – Regional Health Coordinations) of the municipality of São Paulo was presented in the maps to facilitate the visualization of the rates.

The geocoded cases were used to estimate the crude incidence rates of TB-HIV co-infection per triennium and by AD of residence. The average number of new cases per three-year incidence (total number of cases in the triennium divided by three), divided by the resident population of the central year of the triennium, was considered, multiplied by 100,000, resulting in incidence rates per 100,000 inhabitants/year, which were smoothed by the local empirical Bayesian method, with the objective of incorporating the rates of neighboring areas into the analysis, generating risk estimates and controlling random fluctuations ^17^ .

For the spatial dependence analysis of the crude incidence rates of TB-HIV co-infection, we estimated the global Moran index (I) for each triennium. The analysis of local spatial autocorrelation was also performed to identify spatial clusters with greater influence on I, from the estimation of local Moran indices, enabling the construction of three Moran Maps ^18^ , one for each triennium. We used TerraView software, version 4.2.2 and QGIS version 2.16.1 in spatial analyses, and maps to represent the smoothed incidence rates and the Moran Maps were elaborated in the last application.

The profile of case characteristics was described using absolute and relative frequencies, and the individuals were compared according to the type of address (cases with and without fixed residence) using Pearson’s chi-square and Fisher’s exact tests. We considered a 5% significance level (α=0.05) for all analysis.

### Ethical Aspects

All ethical procedures were protected, according to resolution no. 466/2012 of the National Health Council, and the project was approved by the ethics committees of the Faculdade de Saúde Pública of the Universidade de São Paulo (protocol no. 1,609,833) and the Municipality of São Paulo (protocol no. 1,619,747).

## RESULTS

From 2007 to 2015, 51,501 new TB cases were reported in the MSP, according to TBWeb system information. Among these, 6,092 cases had HIV-positive serology, which corresponded to a TB-HIV co-infection ratio of 11.8% in the MSP in the period analyzed. Among all new cases of TB-HIV co-infection included in the study (n = 6,092), 5,609 cases had a defined and fixed address in the FN-TB (92.1%) and 483 cases had no fixed residence (7.9%).

**Figure 1:**
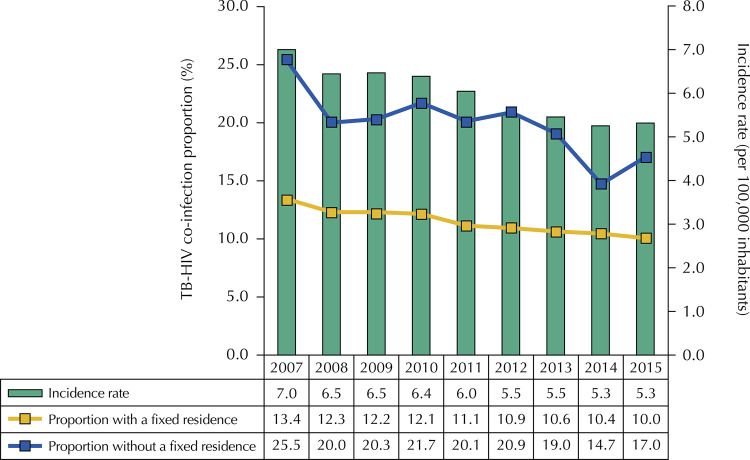
Time series of the proportion of TB-HIV co-infection according to the situation of residence and the incidence rate of co-infection. Municipality of São Paulo, 2007-2015.

Sources: Tuberculosis Patient Control System (TBWeb, 2017); *Fundação Sistema Estadual de Análise de Dados* (Seade, 2017).

A trend of significant decrease in the total proportion of HIV co-infection in new TB cases was identified in the period analyzed, ranging from 13.7% in 2007 to 10.5% in 2015, with an annual decrease of 3.0% (95%CI -3.4 – -2.6). There was a decrease of 3.3% (95%CI -3.7 – -2.8) in the proportion between individuals with a fixed residence and 4.3% (95%CI -6.8 – -1.7) in the population without a fixed residence. The analysis of the temporal trend of the incidence rate of TB-HIV co-infection also revealed a significant decrease in the city of São Paulo, with a decrease of 3.6% per year (95%CI -4.4% – -2.7%), ranging from 7.0 new cases per 100,000 inhabitants in 2007 to 5.3 in 2015 ( Figure 1 ).

**Figure 2:**
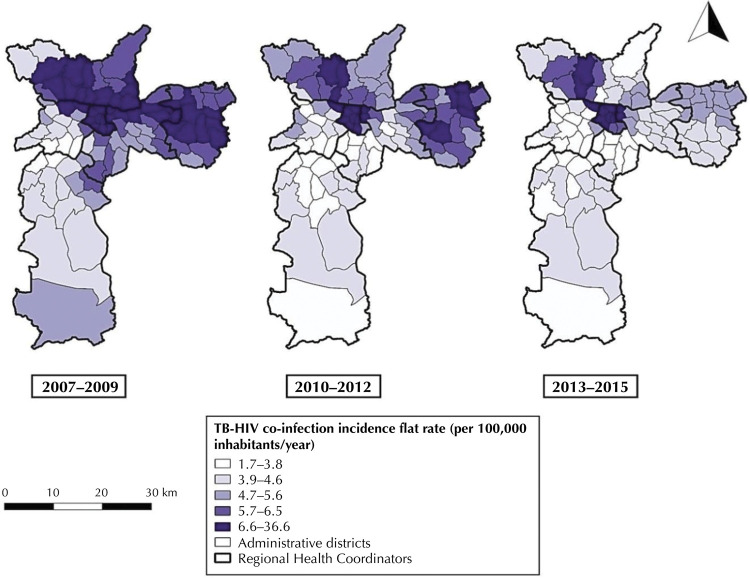
Spatial distribution of TB-HIV co-infection incidence rates flattened by the local empirical Bayesian method, according to the administrative district of residence. Municipality of São Paulo, 2007-2015.

Sources: Coordination of Epidemiology and Information of the Municipal Health Department of São Paulo (CEInfo, 2012); *Centro de Estudos da Metrópole* (CEM, 2016); Tuberculosis Patient Control System (TBWeb, 2017); *Fundação Sistema Estadual de Análise de Dados* (Seade, 2017)

We could geolocate 5,595 (91.8%) of TB-HIV co-infection cases, which were classified according to the AD of residence and the triennium of incidence (2007-2009, 2010–2012, 2013-2015), enabling the construction of maps of the smoothed incidence rates of TB-HIV co-infection ( Figure 2 ). We observed a heterogeneity of spatial distribution, with concentration of cases, especially in AD of the CRS Center, North, Southeast and East in the first triennium (2007–2009); CRS Center, North and East in the second triennium (2010–2012); and remarkable concentration, especially in the CRS Center and North in the third and last triennium (2013–2015).

**Figure 3:**
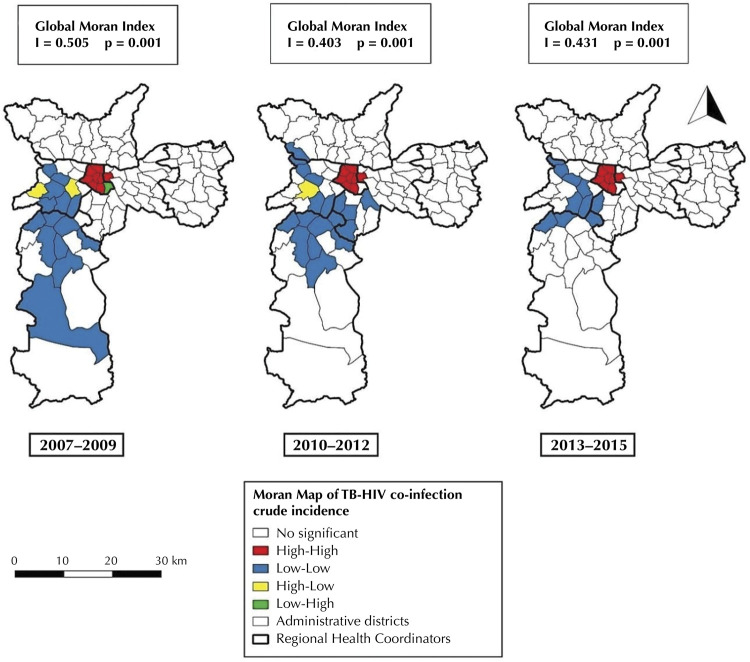
Moran Maps of TB-HIV co-infection incidence rates, according to the administrative district of residence and triennium, with their respective Global Moran indexes (I). Municipality of São Paulo, 2007-2015.

Sources: Coordination of Epidemiology and Information of the Municipal Health Department of São Paulo (CEInfo, 2012); *Centro de Estudos da Metrópole* (CEM, 2016); Tuberculosis Patient Control System (TBWeb, 2017); *Fundação Sistema Estadual de Análise de Dados* (Seade, 2017)

The overall Moran index (I) was positive and statistically significant in all three-year periods (2007–2009: I = 0.505 and p = 0.001; 2010–2012: I = 0.403 and p = 0.001; 2013–2015: I = 0.431 and p = 0.001), which indicates a non-random spatial pattern of the disease in the municipality. The incidence of TB-HIV co-infection in the MSP revealed a positive and significant autocorrelation in all periods, indicating the presence of a high-risk cluster in the central region and a cluster of low risk predominant in the CRS West and South ( Figure 3 ).

The profile of new cases of TB-HIV co-infection has been described according to sociodemographic, clinical and epidemiological characteristics. Individuals with and without fixed residence were compared, and significant differences were identified regarding their gender, race/color, schooling, form of discovery, clinical form, some associated conditions (alcoholism, smoking and drug addiction) and conclusion of treatment of the cases ( Table 1).

**Table:** Sociodemographic, clinical and epidemiological characteristics of new TB-HIV co-infection cases according to the situation of residence. Municipality of São Paulo, 2007-2015.

Characteristics of cases TB-HIV co-infection	With fixed residence		No fixed residence		Total		
	(n = 5.609)		(n = 483)		(n = 6.092)		
	n	%	n	%	n	%	
Gender							< 0.001
Female	1,601	28.5	83	17.2	1,684	27.6	
Male	4,008	71.5	400	82.8	4,408	72.4	
Race/color							< 0.001
White	2,284	40.7	118	24.4	2,402	39.4	
Black or brown	2,170	38.7	247	51.1	2,417	39.7	
Others	45	0.8	02	0.4	47	0.8	
Information ignored	1,110	19.8	116	24.0	1,226	20.1	
Age group							0.087
0 to 19 years old	146	2.6	04	0.8	150	2.5	
20 to 39 years old	2,856	50.9	253	52.4	3,109	51.0	
40 to 59 years old	2,411	43.0	210	43.5	2,621	43.0	
60 years or older	190	3.4	13	2.7	203	3.3	
Information ignored	06	0.1	03	0.6	09	0.1	
Years of schooling							< 0.001
None	93	1.7	17	3.5	110	1.8	
From 1 to 7 years	1,729	30.8	159	32.9	1,888	31.0	
8 years or more	2,280	40.7	108	22.4	2,388	39.2	
Information ignored	1,507	26.9	199	41.2	1,706	28.0	
Diagnostic form/location							< 0.001
Emergency room or hospital	3,686	65.7	301	62.3	3,987	65.4	
Outpatient services	1,683	30.0	118	24.4	1,801	29.6	
Diagnostic after death	140	2.5	26	5.4	166	2.7	
Active search or contact investigation	58	1.0	35	7.2	93	1.5	
Information ignored	42	0.7	03	0.6	45	0.7	
Clinical form ^a^							< 0.001
Pulmonary	3,202	57.1	362	75.1	3,564	58.5	
Extrapulmonary	1,337	23.8	54	11.2	1,391	22.8	
Pulmonary and extrapulmonary or disseminated	1,070	19.1	66	13.7	1,136	18.7	
Associated diabetes	107	1.9	12	2.5	119	2.0	0.379
Associated mental disorder	58	1.0	09	1.9	67	1.1	0.094
Associated alcoholism	539	9.6	142	29.4	681	11.2	< 0.001
Associated smoking	146	2.6	28	5.8	174	2.9	< 0.001
Associated drug addiction	544	9.7	154	31.9	698	11.5	< 0.001
Treatment conclusion ^b^							< 0.001
Cure	3,018	55.5	145	32.7	3,163	53.8	
Death	1,212	22.3	111	25.1	1,323	22.5	
Drop out	1,043	19.2	173	39.1	1,216	20.7	
Failure or resistance	41	0.8	-	0.0	41	0.7	
Transfer to another state/country	58	1.1	05	1.1	63	1.1	
Treatment unconcluded or ignored	65	1.2	09	2.0	74	1.3	

^a^ A case with ignored information about the clinical form was not included (n = 1).

^b^ Cases without information on the initiation of treatment were excluded (n = 212).

Sources: Tuberculosis Patient Control System (TBWeb, 2017); *Fundação Sistema Estadual de Análise de Dados* (Seade, 2017).

## DISCUSSION

The decrease in the incidence of TB-HIV co-infection in the period analyzed shows an important advance in the control of TB and, especially, in HIV control, related to the timely introduction of antiretroviral therapy (ART) and improved access to reference services ^5 , 19 , 20^ . However, aspects related to the diagnosis of TB and treatment conclusion, with emphasis on the large number of diagnoses made in hospital services and the high rates of drop out and death reflect the difficulties in an active search for cases, early diagnosis and effective treatment of TB-HIV co-infection. Spatial analysis allowed the identification of areas with high incidence, which should be a priority for control interventions at the individual and collective level. Some populations are even more affected by co-infection, such as the population without fixed residence, being essential the elaboration of control strategies that consider the singular vulnerabilities of these individuals, thus facilitating their access to health care.

The understanding of the dynamics of TB-HIV co-infection requires timely HIV testing of all individuals diagnosed with TB, as recommended in Brazil ^21^ . The expansion of testing in the MSP in recent years indicates a better organization of TB and HIV/AIDS care services and the improvement of TB and HIV/AIDS control actions ^5 , 15 , 19^ .

The significant decline in the proportion of TB cases co-infected with HIV and in the incidence rate of co-infection identified in our study is consistent with morbidity data for TB and HIV/AIDS in the MSP ^15^ . In recent decades, Brazil, as well as the Americas and the world, has tended to decrease TB incidence and mortality rates ^22^ , as well as a slight reduction in the incidence of TB in the period from 2006 to 2013 in the MSP ^19^ . From this perspective, HIV/aids incidence rates also decreased in MSP in recent years ^20 , 23^ , associated with increased access to HIV infection diagnosis and ART ^24 , 25^ .

The presence of HIV co-infection, about twice higher in TB cases without fixed residence than in the population with fixed residence, may be a reflection of the impact of the HIV/AIDS epidemic in this first group, whose prevalence of HIV infection is 4.9%, whereas the Brazilian population has a rate of 0.6% in the same period ^26^ . In addition to so many other vulnerabilities and conditions of social exclusion, the HIV-infected homeless population has an extremely high risk of TB illness and poor access to health care ^21 , 27^ .

In the TBWeb information system, the main source of data in our study, the entry of the patient’s address data is standardized and free typing is not allowed, and there is as reference a database of patios, thus conditioning the entry of information and increasing the accuracy of case geocoding ^28^ . The use of spatial analysis tools allowed the description of the dispersion of the disease in the territory and the identification of clusters of cases, which can be observed in the maps of figures 2 and 3. Spatial dependence follows the principle that most occurrences have a relationship that depends on the distance, that is, their distribution in the territory ^18^ . The statistically significant global Moran index revealed the existence of positive spatial autocorrelation in the incidence of TB-HIV co-infection, and the local Moran index allowed the delimitation of spatial clusters.

When formulating prevention and control measures for TB-HIV co-infection, the determining aspects of HIV transmission and control in large urban centers should be considered. Some studies have described the patterns of spatial distribution of HIV/AIDS in the MSP ^20 , 23^ , which are similar to that observed in TB-HIV co-infection in our study. TB and HIV/AIDS control programs must configure collaborative actions, both in expanding access to HIV infection diagnosis and timely introduction of ART, as well as in TB prevention through the investigation and treatment of latent infection, in addition to focusing on the groups most vulnerable to infection and illness ^1 , 5 , 21^ .

The geospatial pattern of TB in São Paulo, which reveals strongly affected areas, such as the city center and peripheral areas of the North and East regions ^15 , 19^ , also approaches the spatial distribution of the incidence rates of TB-HIV co-infection observed in our study. The contact with people with the active disease is an essential risk factor for TB infection, i.e., individuals that live or work in environments with high rates of the disease are at higher risk of exposure to bacillus ^16^ . In this sense, knowing the areas with high incidence of TB can contribute to the active search of cases and the breaking of the transmission chain; in the case of populations at higher risk, such as individuals living with HIV/AIDS, this becomes even more necessary ^7^ .

A recent study conducted in Uganda ^29^ identified space clusters of each disease (TB and HIV/AIDS) and TB-HIV co-infection, and concluded that TB rates were positively influenced in the territory by HIV rates and vice versa, that is, it would be necessary to simultaneously approach both diseases for its effective management. Another analysis conducted in Kenya ^30^ also identified a heterogeneous concentration of TB-HIV co-infection cases in the country and stressed the importance of more focused interventions in these regions for better resource allocation.

São Paulo is the most populous city in Brazil ^13^ and has a high population density, especially the most central areas, which present an important urban agglomeration, whether of residents, workers, health units and public transport users, which causes intense people flow, in addition to a greater possibility of transmission of diseases such as TB ^6 , 31^ . In our study, all AD that are part of the high-risk cluster for TB-HIV co-infection have high demographic densities ^32^ , an aspect that has been related to high TB rates in Brazil ^33^ .

When studying a health problem with strong and recognized social determination such as TB, spatial analysis can contribute to the understanding of the health situation of individuals, since the territory goes beyond the meaning of a purely geographical space, also reflecting its insertion in society and its potentials of coping in the health-disease process ^9 , 18^ . However, in Brazilian studies ^34 , 35^ , high TB-HIV co-infection rates were identified also in regions with good socioeconomic indicators, which differs from the traditional panorama found in TB, which mainly affects populations with strong social exclusion. An indicator commonly used to assess the level of development of countries or regions is the human development index. This index, which varies between 0 and 1, is called municipal human development index (MHDI) when used on a municipal scale, composed of three dimensions: longevity, education and income ^36^ . When analyzing the regions of the spatial cluster at high risk for TB-HIV co-infection, we observe that they present high human development and even very high development when only the dimensions of income and longevity are evaluated ^36^ . This may indicate a differentiated social context in the regions most impacted by TB-HIV co-infection, a pattern that is different from that found in the literature in relation to TB ^33^ . Understanding the aspects that determine the concentration of TB-HIV co-infection cases in the MSP can contribute to TB control in the regions at greatest risk and facilitate the formulation of health policies for a more effective organization of TB and HIV/AIDS care services.

The sociodemographic profile of the co-infected population shows the prevalence of men and of people in economically active age ^7 , 37^ . In our study, the population without fixed residence presented a two-fold proportion of black or brown people when compared with white people, corroborating other studies on TB and HIV/AIDS in homeless individuals ^19 , 26 , 38^ . Racial inequalities are determinants of health inequality, since they affect social relationships, self-esteem and access to health care. The low education of the homeless population and an important portion of individuals with fixed residence have already been described in studies on TB-HIV co-infection and may have an impact on understanding the aspects related to the disease and treatment ^7 , 38^ .

The diagnosis, mostly made in emergency services, may reflect advanced stages of the disease, when, finally, the diagnosis is made and treatment is initiated. Frequently, HIV infection is diagnosed concomitantly with TB, implying a huge impact on the lives of these individuals, who, in addition to dealing with two debilitating infectious diseases, each with its specific complexity and treatment, already have a lower chance of cure than individuals not infected with HIV ^39^ . The portion diagnosed after death, especially among individuals without a fixed residence, shows the great difficulty of access to a health service experienced by this population ^26 , 27^ , who dies without a diagnosis and the opportunity to treat the disease.

The predominance of the pulmonary form, present in almost 90% of the co-infected population without fixed residence, is related to the higher risk of transmission, which is increased by aspects such as environmental exposure, precarious accommodation and food conditions and situations of agglomeration ^27^ . Nevertheless, the extrapulmonary form also presented relevant magnitude. In fact, in individuals with important immunological impairment and advanced AIDS, extrapulmonary forms of TB are more common ^7 , 37 , 40^ . However, the adequate use of ART allows the maintenance of immunocompetence and is associated with a decrease in the incidence of TB ^40^ .

In Brazil, care for people living with HIV/AIDS is decentralized, being ideally conducted in primary care and specialized care services (SAE), from the diagnosis of infection, introduction and monitoring of ART to the prevention and treatment of associated diseases ^5 , 21^ . In this sense, the investigation of TB in all care to individuals with HIV/AIDS is a primary action for the timely diagnosis of patients with active TB, being useful also in the prevention of TB illness, since it enables the diagnosis and treatment of latent infection ^41^ .

Alcoholism and drug addiction were observed in an important portion of the population, especially among individuals on the streets. A study conducted in Lima, Peru ^37^ , showed a higher frequency of use of these substances in co-infected individuals than in HIV-negative TB cases. The literature also suggests that the use of psychoactive substances is related to the development and transmission of TB, since they decrease individuals’ immunological defense ^16^ . Moreover, the association between the harmful use of alcohol and other drugs and unfavorable treatment outcomes, such as drop out, is remarkable ^16 , 27 , 38^ . The importance of investigating these diseases in diagnosis and during treatment is emphasized, as a way to identify the difficulties for the treatment and strengthen the bond with the health team.

In our study, a total of 97.7% of the cases had the treatment outcome registered in the FN-TB and, in this group, cure rates were below the recommended (≥ 85%) and lower than those of individuals not infected with HIV in the same social context ^19^ . In the association between TB and HIV, the risk of death during treatment is 3 to 19 times higher than in individuals not infected with HIV ^39^ . Previous studies also corroborate the lowest proportions of cure in co-infected patients ^37 , 38 , 40 , 42^ , and in this perspective, more robust health policies are needed, focusing on the population living with HIV and suffering from TB, with integrated strategies that contribute to the early diagnosis, proper management of diseases and treatment adherence.

In individuals co-infected with HIV, directly observed treatment (DOT) has been shown to be even more necessary, due to the complexity of the diseases and the possibility of drug interactions with ART ^2 , 21^ , and should be offered to all TB patients ^21^ . In 2017, however, among the co-infected cases that used ART, only 22.1% were in DOT and, in the group that did not use ART, the proportion that treated TB with supervision was even lower (13.9%) ^5^ . Epidemiological surveillance plays a fundamental role in the articulation with the care network and in case monitoring, which may contribute to timely interventions of the health team and to the expansion of DOT, strengthening the control of the disease in the territory ^5^ .

In the population without fixed residence, treatment outcomes are even more unfavorable, with almost twice the dropout rate of the population with fixed residence and the cure achieved by only one third of the individuals undergoing treatment, findings corroborated by other studies ^27 , 38^ . Considering the numerous social, individual and programmatic vulnerabilities experienced by these individuals some can be highlighted, such as poor daily feeding, alcohol abuse and other psychoactive substances, difficulties in accessing and referring to a health service and stigma and prejudice towards society ^26 , 27 , 38^ , which can hinder access to health care and contribute to low treatment.

In this sense, differentiated strategies are essential to deal with the challenge of treatment adherence by homeless population. Intersectoral articulation, with partnerships with social assistance, social organizations and the support of civil society itself, is essential for the success of actions ^15^ . The relevance of DOT to enhance the treatment through approximation with the health team and the offer of social incentives, being important for these individuals that experience a singular and challenging social context for the treatment and disease control ^27 , 38 , 43^ . The homeless population should be considered in the formulation of specific policies to control TB-HIV co-infection. Health professionals and services must be prepared to meet their demands, not only looking at clinical aspects, but also integrating the social approach and the strengthening of citizenship.

Ecological studies have some limitations. It is not possible to affirm that the conclusions occur similarly at the individual level with an aggregate data analysis ^44^ . The AD as a spatial unit of analysis presents a large territorial extension and with heterogeneity of characteristics; however, since it is an administrative division, its use can facilitate decision-making during the planning and organization of control actions focusing on priority areas.

Moreover, the data used are secondary, coming from an epidemiological surveillance information system powered by case notifications, and may contain filling failures, outdated data and lack of information; therefore, analyses should be carefully interpreted. Incomplete information can hinder the real understanding of who is getting sick and, thus, interfere in the planning of control actions ^45^ , which can be modified and improved through education, either during academic training or in the continuous training of professionals and by the qualification of information by probabilistic relationships among the databases used in epidemiological surveillance ^5^ .

The knowledge of the use of ART could provide more elements for understanding the clinical evolution of these individuals. This variable was only included in the FN-TB in 2016, allowing the analysis of the individuals included in our study; however, it may add important aspects in future analyses of this disease ^5^ . Finally, the results of our study can help in the organization of health care services and in the improvement of collaborative activities by TB and HIV/AIDS control programs, strengthening actions for prevention, diagnosis and treatment of TB-HIV co-infection.

## References

[1] 1. World Health Organization. Global tuberculosis report 2019. Geneva: WHO; 2019 [cited 2020 Mar 9]. Available from: https://apps.who.int/iris/bitstream/handle/10665/329368/9789241565714-eng.pdf?ua=1

[2] 2. Reid A, Scano F, Getahun H, Williams B, Dye C, Nunn P, et al. Towards universal access to HIV prevention, treatment, care, and support: the role of tuberculosis/HIV collaboration. Lancet Infect Dis. 2006;6(8):483-95. htps://doi.org/10.1016/S1473-3099(06)70549-7 10.1016/S1473-3099(06)70549-7 16870527

[3] 3. Raviglione M, Sulis G. Tuberculosis 2015: burden, challenges and strategy for control and elimination. Infect Dis Rep. 2016;8(2):6570. 10.4081/idr.2016.6570 PMC4927938 27403269

[4] 4. Friedland G, Churchyard GJ, Nardell E. Tuberculosis and HIV coinfection: current state of knowledge and research priorities. J Infect Dis. 2007;196 Suppl 1:S1-3. 10.1086/518667 17624818

[5] 5. Ministério da Saúde (BR). Panorama epidemiológico da coinfecção TB-HIV no Brasil 2019. Brasília, DF; 2019 [cited 2020 Mar 9]. Available from: https://portalarquivos2.saude.gov.br/images/pdf/2019/outubro/01/Boletim-tuberculose-2019.pdf

[6] 6. Rodrigues-Jr AL, Ruffino-Netto A, Castilho EA. Distribuição espacial da co-infecção M. tuberculosis/HIV no Estado de São Paulo, 1991-2001. Rev Saude Publica. 2006;40(2):265-70. 10.1590/S0034-89102006000200012 16583037

[7] 7. Brunello MEF, Chiaravalloti Neto F, Arcêncio RA, Andrade RLP, Magnabosco GT, Villa TCS. Áreas de vulnerabilidade para co-infecção HIV-aids/TB em Ribeirão Preto, SP. Rev Saude Publica. 2011;45(3):556-63. 10.1590/S0034-89102011005000018 21484011

[8] 8. Vendramini SHF, Santos NSGM, Santos MLSG, Chiaravalloti-Neto F, Ponce MAZ, Gazetta CE, et al. Análise espacial da co-infecção tuberculose/HIV: relação com níveis socioeconômicos em município do sudeste do Brasil. Rev Soc Bras Med Trop. 2010;43(5):536-41. 10.1590/S0037-86822010000500013 21085865

[9] 9. Rodrigues-Júnior AL, Ruffino-Netto A, Castilho EA. Spatial distribution of the human development index, HIV infection and AIDS-tuberculosis comorbidity: Brazil, 1982 - 2007. Rev Bras Epidemiol. 2014;17 Supl 2:204-15. 10.1590/1809-4503201400060017 25409649

[10] 10. Chan-yeung M, Yeh AGO, Tam CM, Kam KM, Leung CC, Yew WW, et al. Socio-demographic and geographic indicators and distribution of tuberculosis in Hong Kong: a spatial analysis. Int J Tuberc Lung Dis. 2005;9(12):1320-6. 16466053

[11] 11. Antunes JLF, Cardoso MRA. Uso da análise de séries temporais em estudos epidemiológicos. Epidemiol Serv Saude. 2015;24(3):565-76. 10.5123/S1679-49742015000300024

[12] 12. Villaça F. São Paulo: segregação urbana e desigualdade. Estud Av. 2011;25(71):37-58. 10.1590/S0103-40142011000100004

[13] 13. Instituto Brasileiro de Geografia e Estatística. Censo Demográfico: microdados. Rio de Janeiro: IBGE; 2010 [cited 2020 Mar 9]. Available from: https://www.ibge.gov.br/estatisticas/sociais/populacao/9662-censo-demografico-2010.html?edicao=9748&t=resultados

[14] 14. Fundação Sistema Estadual de Análise de Dados. Sistema SEADE de projeções populacionais. São Paulo: SEADE; 2017 [cited 2020 Mar 9]. Available from: https://produtos.seade.gov.br/produtos/projpop/

[15] 15. Secretaria Municipal de Saúde de São Paulo, Coordenação de Vigilância em Saúde, Centro de Controle de Doenças. Programa Municipal de Controle da Tuberculose. Bol TB Cidade de São Paulo. 2016 [cited 2020 Mar 9]. Available from: https://www.prefeitura.sp.gov.br/cidade/secretarias/upload/saude/vigilancia_em_saude/arquivos/boletimTb_2016_menor.pdf

[16] 16. Lönnroth K, Jaramillo E, Williams BG, Dye C, Raviglione M. Drivers of tuberculosis epidemics: the role of risk factors and social determinants. Soc Sci Med. 2009;68(12):2240-6. 10.1016/j.socscimed.2009.03.041 19394122

[17] 17. Yamamura M, Freitas IM, Santos Neto M, Chiaravalloti Neto F, Popolin MAP, Arroyo LH, et al. Análise espacial das internações evitáveis por tuberculose em Ribeirão Preto, SP (2006-2012). Rev Saude Publica. 2016;50:20. 10.1590/S1518-8787.2016050006049 PMC4902087 27191156

[18] 18. Druck S, Carvalho MS, Câmara G, Monteiro AMV, editores. Análise espacial de dados geográficos. Planaltina, DF: Embrapa; 2004 [cited 2020 Mar 9]. Available from: http://www.dpi.inpe.br/gilberto/livro/analise/

[19] 19. Pinto PFPS, Silveira C, Rujula MJP, Chiaravalloti Neto F, Ribeiro MCSA. Epidemiological profile of tuberculosis in São Paulo municipality from 2006 to 2013. Rev Bras Epidemiol. 2017;20(3):549-57. 10.1590/1980-5497201700030016 29160445

[20] 20. Pellini ACG. Evolução da epidemia de Aids no município de São Paulo - 1980 a 2012: uma análise espacial com múltiplas abordagens [tese]. São Paulo: Faculdade de Saúde Pública da USP; 2016 [cited 2020 Mar 9]. Available from: http://www.teses.usp.br/teses/disponiveis/6/6132/tde-09122016-144047/pt-br.php

[21] 21. Ministério da Saúde (BR). Recomendações para o manejo da coinfecção TB-HIV em serviços de atenção especializada a pessoas vivendo com HIV/AIDS. Brasília, DF; 2013 [cited 2020 Mar 9]. Available from: http://bvsms.saude.gov.br/bvs/publicacoes/recomendacoes_manejo_coinfeccao_tb_hiv.pdf

[22] 22. Guimarães RM, Lobo ADP, Siqueira EA, Borges TFF, Melo SCC. Tuberculose, HIV e pobreza: tendência temporal no Brasil, Américas e mundo. J Bras Pneumol. 2012;38(4):511-7. 10.1590/S1806-37132012000400014 22964936

[23] 23. Aguiar BS. Análise espacial e espaço temporal da Aids no município de São Paulo entre 2001 e 2010 [dissertação]. São Paulo: Faculdade de Saúde Pública da USP; 2013 [cited 2020 Mar 9]. Available from: http://www.teses.usp.br/teses/disponiveis/6/6132/tde-11112013-135905/pt-br.php

[24] 24. Tancredi MV, Waldman EA. Survival of AIDS patients in Sao Paulo-Brazil in the pre- and post-HAART eras: a cohort study. BMC Infect Dis. 2014;14:599. 10.1186/s12879-014-0599-8 PMC4247874 25398533

[25] 25. Dourado I, Veras MASM, Barreira D, Brito AM. Tendências da epidemia de Aids no Brasil após a terapia anti-retroviral. Rev Saude Publica. 2006;40 Supl:9-17. 10.1590/S0034-89102006000800003 16729154

[26] 26. Grangeiro A, Holcman MM, Onaga ET, Alencar HDR, Placco ALN, Teixeira PR. Prevalência e vulnerabilidade à infecção pelo HIV de moradores de rua em São Paulo, SP. Rev Saude Publica. 2012;46(4):674-84. 10.1590/S0034-89102012005000037 22715004

[27] 27. Oliveira AAV, Oliveira RCC, Barbosa KKS, Mendonça AVM, Sousa MF, Sá LD. The access of the homeless persons with tuberculosis to the health care: an integrative review. Int Arch Med. 2017;10. 10.3823/2384

[28] 28. Magalhães MAFM, Matos VP, Medronho RA. Avaliação do dado sobre endereço no Sistema de Informação de Agravos de Notificação, utilizando georreferenciamento em nível local de casos de tuberculose, por dois métodos no município do Rio de Janeiro. Cad Saude Coletiva. 2014;22(2):192-9. 10.1590/1414-462X201400020013

[29] 29. Aturinde A, Farnaghi M, Pilesjö P, Mansourian A. Spatial analysis of HIV-TB co-clustering in Uganda. BMC Infect Dis. 2019;19(1):612. 10.1186/s12879-019-4246-2 PMC6625059 31299907

[30] 30. Otiende V, Achia T, Mwambi H. Bayesian modeling of spatiotemporal patterns of TB-HIV co-infection risk in Kenya. BMC Infect Dis. 2019;19(1):902. 10.1186/s12879-019-4540-z PMC6819548 31660883

[31] 31. Touray K, Adetifa IM, Jallow A, Rigby J, Jeffries D, Cheung YB, et al. Spatial analysis of tuberculosis in an urban west African setting: is there evidence of clustering? Trop Med Int Health. 2010;15(6):664-72. 10.1111/j.1365-3156.2010.02533.x 20406427

[32] 32. Secretaria Municipal de Desenvolvimento Urbano de São Paulo. Demografia: tabelas: população recenseada, taxas de crescimento populacional e densidade demográfica - Município de São Paulo, Subprefeituras e Distritos Municipais, 1980, 1991, 2000 e 2010. São Paulo: Infocidade; 2010 [cited 2020 Mar 9]. Available from: https://www.prefeitura.sp.gov.br/cidade/secretarias/urbanismo/dados_estatisticos/info_cidade/demografia/index.php?p=260265

[33] 33. Harling G, Castro MC. A spatial analysis of social and economic determinants of tuberculosis in Brazil. Health Place. 2014;25:56-67. 10.1016/j.healthplace.2013.10.008 24269879

[34] 34. Peruhype RC, Acosta LMW, Ruffino Neto A, Oliveira MMC, Palha PF. Distribuição da tuberculose em Porto Alegre: análise da magnitude e coinfecção tuberculose-HIV. Rev Esc Enferm USP. 2014;48(6):1035-43. 10.1590/S0080-623420140000700011 25626503

[35] 35. Souza AG, Fukushima M, Pereira TB, Picanço MRA, Tatsch JFS, Miranda Junior UJP. Contextualização de aspectos sociais da coinfecção TB/HIV no Distrito Federal. Rev Eletr Gestao Saude. 2013 [cited 2020 Mar 9];4(1):1516-29. Available from: https://periodicos.unb.br/index.php/rgs/article/view/178

[36] 36. Gonçalves AF, Maeda MT. IDH e a dinâmica intraurbana na cidade de São Paulo. In: Marguti BO, Costa MA, Favarão CB, organizadores. Territórios em números: insumos para políticas públicas a partir da análise do IDHM e do IVS de UDHs e regiões metropolitanas brasileiras. Brasília, DF: Instituto de Pesquisa Econômica Aplicada; 2017. p.171-91.

[37] 37. Velásquez GE, Cegielski JP, Murray MB, Yagui MJA, Asencios LL, Bayona JN, et al. Impact of HIV on mortality among patients treated for tuberculosis in Lima, Peru: a prospective cohort study. BMC Infect Dis. 2016;16:45. 10.1186/s12879-016-1375-8 PMC4736097 26831140

[38] 38. Ranzani OT, Carvalho CRR, Waldman EA, Rodrigues LC. The impact of being homeless on the unsuccessful outcome of treatment of pulmonary TB in São Paulo State, Brazil. BMC Med. 2016;14:41. 10.1186/s12916-016-0584-8 PMC4804546 27006009

[39] 39. Marks SM, Magee E, Robison V. Patients diagnosed with tuberculosis at death or who died during therapy: association with the human immunodeficiency virus. Int J Tuberc Lung Dis. 2011;15(4):465-70. 10.5588/ijtld.10.0259 PMC5451101 21396204

[40] 40. Kwan CK, Ernst JD. HIV and tuberculosis: a deadly human syndemic. Clin Microbiol Rev. 2011;24(2):351-76. 10.1128/CMR.00042-10 PMC3122491 21482729

[41] 41. Golub JE, Cohn S, Saraceni V, Cavalcante SC, Pacheco AG, Moulton LH, et al. Long-term protection from isoniazid preventive therapy for tuberculosis in HIV-infected patients in a medium-burden tuberculosis setting: the TB/HIV in Rio (THRio) study. Clin Infect Dis. 2015;60(4):639-45. 10.1093/cid/ciu849 PMC4366579 25365974

[42] 42. Prado TN, Miranda AE, Souza FM, Dias ES, Sousa LKF, Arakaki-Sanchez D, et al. Factors associated with tuberculosis by HIV status in the Brazilian National Surveillance System: a cross sectional study. BMC Infect Dis. 2014;14:415. 10.1186/1471-2334-14-415 PMC4122782 25066655

[43] 43. Alecrim TFA, Mitano F, Reis AA, Roos CM, Palha PF, Protti-Zanatta ST. Experiência dos profissionais de saúde no cuidado da pessoa com tuberculose em situação de rua. Rev Esc Enferm USP. 2016;50(5):808-15. 10.1590/s0080-623420160000600014 27982400

[44] 44. Morgenstern H. Ecologic studies in epidemiology: concepts, principles, and methods. Annu Rev Public Health. 1995;16:61-81. https://doi.rg/10.1146/annurev.pu.16.050195.000425 10.1146/annurev.pu.16.050195.000425 7639884

[45] 45. Moreira CMM, Maciel ELN. Completude dos dados do Programa de Controle da Tuberculose no Sistema de Informação de Agravos de Notificação no Estado do Espírito Santo, Brasil: uma análise do período de 2001 a 2005. J Bras Pneumol. 2008;34(4):225-9. 10.1590/S1806-37132008000400007

