# Supplementary material for: TB-HIV co-infection: spatial and temporal distribution in the largest Brazilian metropolis
Source: Rev Saude Publica. 2020 Oct 28;54:e112. doi: 10.11606/s1518-8787.2020054002108 (PMC7593020; doi:10.11606/s1518-8787.2020054002108)
Supplement: Translation

# Translation: Coinfecção TB-HIV: distribuição espacial e temporal na maior metrópole brasileira

**Authors:** Roberta Figueiredo Cavalin, Alessandra Cristina Guedes Pellini, Regina Rocha Gomes de Lemos, Ana Paula Sayuri Sato

**Affiliations:** I Brasil Universidade de São Paulo. Faculdade de Saúde Pública. Programa de Pós-Graduação em Saúde Pública. São Paulo, SP, Brasil; II Brasil Universidade Nove de Julho. Faculdade de Medicina. Diretoria de Ciências Médicas. São Paulo, SP, Brasil; III Brasil Secretaria Municipal da Saúde. Coordenação de Vigilância em Saúde de São Paulo. Programa Municipal de Controle da Tuberculose. São Paulo, SP, Brasil; IV Brasil Universidade de São Paulo. Faculdade de Saúde Pública. Departamento de Epidemiologia. São Paulo, SP, Brasil

**Keywords:** Tuberculose, epidemiologia, Infecções por HIV, epidemiologia, Análise Espacial, Estudos de Séries Temporais

## Abstract

**OBJETIVO:**

Descrever a distribuição espacial e temporal da coinfecção TB-HIV, assim como o perfil das características da população coinfectada no município de São Paulo.

**MÉTODOS:**

Estudo ecológico e de série temporal com dados do Sistema de Controle de Pacientes com Tuberculose (TBWeb), incluindo todos os casos novos de tuberculose coinfectados pelo HIV residentes no município no período de 2007 a 2015. Tendências temporais do agravo foram analisadas por regressão de Prais-Winsten. Os casos foram geocodificados pelo endereço de residência para a elaboração de mapas com as taxas de incidência suavizadas pelo método bayesiano empírico local. Os índices de Moran global e local avaliaram a autocorrelação espacial. O perfil dos indivíduos foi descrito e as características dos casos com e sem residência fixa foram comparadas pelos testes de qui-quadrado ou exato de Fisher.

**RESULTADOS:**

Foram analisados 6.092 casos novos de coinfecção TB-HIV (5.609 com residência fixa e 483 sem residência fixa). A proporção de coinfecção TB-HIV variou de 10,5% a 13,7%, com queda de 3,0% ao ano (IC95% -3,4 – -2,6), e foi maior nos indivíduos sem residência fixa em todo o período. As taxas de incidência apresentaram diminuição de 3,6% ao ano (IC95% -4,4% – -2,7%), declinando de 7,0 para 5,3 por 100 mil habitantes/ano. A coinfecção apresentou autocorrelação espacial positiva e significativa, com padrão espacial heterogêneo e um aglomerado de alto risco na região central do município. A cura foi alcançada em 55,5% dos casos com residência fixa e em 32,7% daqueles sem residência.

**CONCLUSÕES:**

Os dados indicam um importante avanço no controle da coinfecção TB-HIV no período analisado. Todavia, foram identificadas áreas e populações que se apresentaram desigualmente afetadas pelo agravo, e que devem ser priorizadas no aprimoramento das ações de prevenção e controle da coinfecção.

## INTRODUÇÃO

Embora seja uma das doenças infecciosas mais antigas da história da humanidade, a tuberculose (TB) ainda representa um grande desafio à saúde pública global, provocando o adoecimento de 10,0 milhões de pessoas e 1,4 milhões de mortes em 2018 ^1^ . Seguida pela síndrome da imunodeficiência adquirida (aids), a TB é a mais importante causa de mortalidade por agente infeccioso no mundo ^1^ .

Com início na década de 1980, a epidemia de aids impactou fortemente o perfil epidemiológico e o controle da TB. O comprometimento imunológico causado pelo HIV/aids favorece a multiplicação do *M. tuberculosis* e o adoecimento por TB ^2^ , e a infecção pelo HIV é o fator de risco mais importante para a TB ativa, com risco 19 vezes maior na população infectada pelo vírus em comparação à população geral ^1^ . A sinergia entre os agravos também é observada na mortalidade associada ao HIV, sendo a TB a principal causa de morte entre as pessoas vivendo com HIV/aids ^3^ .

No ano de 2018, estima-se que cerca de 8,6% de todos os casos de TB no mundo eram HIV-positivos, totalizando 862 mil pessoas acometidas pela coinfecção ^1^ . A coinfecção TB-HIV aflige de forma contundente as pessoas que vivem em condições precárias de vida, que sofrem com a falta de recursos para a prevenção, o diagnóstico, o tratamento e o controle, tanto da TB quanto do HIV/aids ^1 , 4^ . Observa-se que a coinfecção afeta, sobretudo, regiões subdesenvolvidas e populosas, como algumas áreas do continente africano, em que ela representa mais de 50% dos casos ^1^ .

Em 2017, o Brasil apresentou 11,4% dos casos novos de TB coinfectados pelo vírus HIV, e o estado de São Paulo identificou 9,3% de coinfecção ^5^ . Considerando a classificação da Organização Mundial da Saúde ^1^ , que define os países com maiores cargas de TB, a atual situação epidemiológica do Brasil o enquadra nos contextos de alta carga de TB e do agravo associado ao HIV, sendo, portanto, um dos países prioritários para investimentos em ações de controle.

Apesar da relevância da coinfecção, são ainda escassas as publicações sobre a sua distribuição espacial e temporal no Brasil ^6^ . Os sistemas de informação geográfica (SIG) são valiosas ferramentas para a análise dos dados espaciais na área da saúde, e o seu uso pode contribuir para a vigilância epidemiológica de agravos transmissíveis como a TB e o HIV/aids, pois facilitam o conhecimento da distribuição dos casos no território e permitem investigar os fatores associados à transmissão e identificar áreas prioritárias para intervenções ^10^ . Na área da saúde, a medida de tempo também é muito útil para o entendimento do objeto de estudo. As séries temporais, que são formas de organizar as informações quantitativas no tempo ^11^ , podem ser utilizadas para caracterizar as tendências temporais de agravos que impactam a saúde das populações, assim como para avaliar a efetividade das políticas de controle.

Revelando intensas desigualdades sociais ^12^ , com uma complexa rede de assistência à saúde e a maior densidade demográfica do país ^13^ , o município de São Paulo (MSP) apresenta o maior número de casos de coinfecção TB-HIV no Brasil ^5^ e, portanto, demanda o conhecimento da dinâmica do agravo para promover o aprimoramento das ações de controle. Nesse sentido, o presente estudo propõe descrever a distribuição espacial e temporal da coinfecção TB-HIV, assim como o perfil das características da população coinfectada no MSP.

## MÉTODOS

### Desenho e Área do Estudo

Trata-se de um estudo ecológico com componente analítico e de séries temporais desenvolvido no MSP, localizado na região Sudeste do Brasil, que é o município mais populoso do país, com um total de 11.638.802 habitantes no ano de 2016 e a quase totalidade da população residindo na zona urbana (99,1%) ^14^ . A gestão das ações de controle da TB é realizada pelo Programa Municipal de Controle da Tuberculose (PMCT), de forma descentralizada ^15^ .

### População e Período do Estudo

Foram incluídos todos os casos novos de TB coinfectados pelo vírus HIV residentes no MSP e com incidência no período de 2007 a 2015, exceto aqueles com mudanças de diagnóstico e pacientes privados de liberdade. Definiu-se como “caso com residência fixa” o indivíduo que forneceu um endereço de moradia fixa no momento da notificação da TB, e o caso “sem residência fixa” foi definido como o indivíduo sem endereço de moradia fixa constante na ficha de notificação de TB (FN-TB). Este trabalho analisou separadamente as populações com e sem residência fixa, considerando que as singulares vulnerabilidades que integram a vida dos indivíduos em situação de rua podem influenciar o processo saúde-doença, em especial na TB, doença socialmente determinada ^16^ .

### Fontes de Dados

Os dados dos casos de coinfecção TB-HIV foram extraídos em 5 de junho de 2017 do Sistema de Controle de Pacientes com Tuberculose (TBWeb), da Secretaria de Estado da Saúde de São Paulo, que armazena as informações da FN-TB. A base digital de logradouros e o mapa digital dos distritos administrativos (DA) do MSP foram obtidos do Centro de Estudos da Metrópole (CEM) e da Coordenação de Epidemiologia e Informação da Secretaria Municipal de Saúde de São Paulo (CEInfo). Foram utilizados também os dados relativos à população residente do MSP da Fundação Sistema Estadual de Análise de Dados (Seade).

### Análises

A proporção da coinfecção TB-HIV entre os casos novos de TB foi calculada para cada ano do estudo, utilizando o número total de casos novos de TB e o número de casos com sorologia anti-HIV positiva, conforme as informações do TBWeb. Para o cálculo da taxa anual de incidência, considerou-se o número total de casos novos de coinfecção TB-HIV por ano, dividido pelo total da população residente estimada no meio do mesmo ano, multiplicado por 100.000, resultando em uma taxa de incidência por 100.000 habitantes/ano.

Para verificar a tendência temporal das proporções e das taxas de incidência de coinfecção TB-HIV no município, foram construídos modelos de regressão linear generalizados pelo método de Prais-Winsten ^11^ . Para estimar quantitativamente as tendências temporais da coinfecção no período analisado, foi utilizada a fórmula de variação percentual anual (do inglês *annual percent change* – APC), assim como o cálculo do intervalo de confiança de 95% (IC95%) ^11^ . Os modelos de regressão linear foram construídos no *software* Stata, versão 12.

Para caracterizar os padrões de distribuição espacial da coinfecção TB-HIV, os indivíduos foram georreferenciados a partir do endereço de residência. A unidade espacial de escolha foi o DA, que é a menor divisão administrativa do MSP. Para todas as análises espaciais deste estudo, os dados foram apresentados conforme os triênios de incidência do agravo (2007–2009, 2010–2012 e 2013–2015), incorporando, assim, a abordagem temporal para a verificação das modificações espaciais ocorridas ao longo do período do estudo e os padrões de difusão do agravo. A divisão das Coordenadorias Regionais de Saúde (CRS) do MSP foi apresentada nos mapas para facilitar a visualização das taxas.

Os casos geocodificados foram utilizados para o cálculo das taxas brutas de incidência de coinfecção TB-HIV por triênio e por DA de residência. Considerou-se o número médio de casos novos por triênio de incidência (número total de casos no triênio dividido por três), dividido pela população residente do ano central do triênio, multiplicado por 100.000, resultando em taxas de incidência por 100.000 habitantes/ano, que foram suavizadas pelo método bayesiano empírico local, com o objetivo de incorporar as taxas das áreas vizinhas na análise, gerando estimativas de risco e controlando flutuações aleatórias ^17^ .

Para a análise de dependência espacial das taxas brutas de incidência de coinfecção TB-HIV, foi calculado o índice de Moran global (I) para cada triênio. A análise da autocorrelação espacial local também foi realizada para identificar aglomerados espaciais com maior influência no I, a partir do cálculo dos índices de Moran locais, possibilitando a construção de três *Moran Maps* ^18^ , um para cada triênio. Os *softwares* TerraView, versão 4.2.2 e QGIS versão 2.16.1 foram utilizados nas análises espaciais, e os mapas para a representação das taxas de incidência suavizadas e os *Moran Maps* foram elaborados nesse último aplicativo.

O perfil de características dos casos foi descrito utilizando frequências absolutas e relativas, e os indivíduos foram comparados segundo o tipo de endereço (casos com e sem residência fixa) utilizando os testes de qui-quadrado de Pearson e exato de Fisher. Para todas as análises do estudo, foi considerando o nível de significância de 5%.

### Aspectos Éticos

Foram resguardados todos os procedimentos éticos, conforme a resolução n⁰ 466/2012 do Conselho Nacional de Saúde, e o projeto foi aprovado pelos comitês de ética da Faculdade de Saúde Pública da Universidade de São Paulo (protocolo n⁰ 1.609.833) e do MSP (protocolo n⁰ 1.619.747).

## RESULTADOS

No período de 2007 a 2015, foram notificados 51.501 casos novos de TB residentes no MSP, segundo as informações do sistema TBWeb. Dentre esses, 6.092 casos apresentavam sorologia positiva para o HIV, o que corresponde a uma proporção de coinfecção TB-HIV de 11,8% no MSP no período analisado. Destaca-se que, dentre todos os casos novos de coinfecção TB-HIV incluídos no estudo (n = 6.092), 5.609 casos apresentavam endereço definido e fixo na FN-TB (92,1%) e 483 casos não tinham residência fixa (7,9%).

Foi identificada uma tendência de queda significativa na proporção total de coinfecção pelo HIV nos casos novos de TB no período analisado, variando de 13,7% em 2007 a 10,5% em 2015, com diminuição anual de 3,0% (IC95% -3,4 – -2,6). Houve decréscimo de 3,3% (IC95% -3,7 – -2,8) na proporção entre os indivíduos com residência fixa e de 4,3% (IC95% -6,8 – -1,7) na população sem residência. A análise de tendência temporal da taxa de incidência da coinfecção TB-HIV também revelou uma queda significante no município de São Paulo, com decréscimo de 3,6% ao ano (IC95% -4,4% – -2,7%), variando de 7,0 casos novos a cada 100 mil habitantes em 2007 para 5,3 em 2015 (Figura 1).

**Figura 1:** Série temporal da proporção de coinfecção TB-HIV segundo situação de residência e da taxa de incidência da coinfecção. Município de São Paulo, 2007–2015.

Fontes: Sistema de Controle de Pacientes com Tuberculose (TBWeb, 2017); Fundação Sistema Estadual de Análise de Dados (Seade, 2017).

Foi possível geolocalizar 5.595 (91,8%) dos casos de coinfecção TB-HIV, que foram classificados conforme o DA de residência e o triênio de incidência (2007–2009, 2010–2012, 2013–2015), possibilitando a construção de mapas das taxas suavizadas de incidência de coinfecção TB-HIV (Figura 2). Observa-se heterogeneidade da distribuição espacial, com concentração de casos, sobretudo, em DA das CRS Centro, Norte, Sudeste e Leste no primeiro triênio (2007–2009); CRS Centro, Norte e Leste no segundo triênio (2010–2012); e notável concentração, sobretudo, nas CRS Centro e Norte no terceiro e último triênio (2013–2015).

**Figura 2:** Distribuição espacial das taxas de incidência de coinfecção TB-HIV suavizadas pelo método bayesiano empírico local, segundo distrito administrativo de residência. Município de São Paulo, 2007–2015.

Fontes: Coordenação de Epidemiologia e Informação da Secretaria Municipal de Saúde de São Paulo (CEInfo, 2012); Centro de Estudos da Metrópole (CEM, 2016); Sistema de Controle de Pacientes com Tuberculose (TBWeb, 2017); Fundação Sistema Estadual de Análise de Dados (Seade, 2017

O índice de Moran global (I) foi positivo e estatisticamente significativo em todos os triênios (2007–2009: I = 0,505 e p = 0,001; 2010–2012: I = 0,403 e p = 0,001; 2013–2015: I = 0,431 e p = 0,001), o que indica um padrão espacial não aleatório do agravo no município. A incidência da coinfecção TB-HIV no MSP revelou autocorrelação positiva e significativa em todos os períodos analisados, indicando a presença de um aglomerado de alto risco na região central e um aglomerado de baixo risco predominante nas CRS Oeste e Sul (Figura 3).

**Figura 3:** *Moran Maps* das taxas de incidência de coinfecção TB-HIV, segundo distrito administrativo de residência e triênio, com seus respectivos índices de Moran globais (I). Município de São Paulo, 2007–2015.

Fontes: Coordenação de Epidemiologia e Informação da Secretaria Municipal de Saúde de São Paulo (CEInfo, 2012); Centro de Estudos da Metrópole (CEM, 2016); Sistema de Controle de Pacientes com Tuberculose (TBWeb, 2017); Fundação Sistema Estadual de Análise de Dados (Seade, 2017).

O perfil dos casos novos de coinfecção TB-HIV foi descrito segundo características sociodemográficas, clínicas e epidemiológicas. Os indivíduos com e sem residência fixa foram comparados, e foram identificadas diferenças significativas em relação ao sexo, raça/cor, escolaridade, forma de descoberta, forma clínica, alguns agravos associados (alcoolismo, tabagismo e drogadição) e encerramento de tratamento dos casos (Tabela 1).

**Tabela 1:** Características sociodemográficas, clínicas e epidemiológicas dos casos novos de coinfecção TB-HIV segundo a situação de residência. Município de São Paulo, 2007–2015.

Características dos casos de coinfecção TB-HIV	Com residência fixa		Sem residência fixa		Total		p
	(n = 5.609)		(n = 483)		(n = 6.092)		
	n	%	n	%	n	%	
Sexo							< 0,001
Feminino	1.601	28,5	83	17,2	1.684	27,6	
Masculino	4.008	71,5	400	82,8	4.408	72,4	
Raça/cor							< 0,001
Branca	2.284	40,7	118	24,4	2.402	39,4	
Preta ou parda	2.170	38,7	247	51,1	2.417	39,7	
Outras	45	0,8	02	0,4	47	0,8	
Informação ignorada	1.110	19,8	116	24,0	1.226	20,1	
Faixa etária							0,087
0 a 19 anos	146	2,6	04	0,8	150	2,5	
20 a 39 anos	2.856	50,9	253	52,4	3.109	51,0	
40 a 59 anos	2.411	43,0	210	43,5	2.621	43,0	
60 anos ou mais	190	3,4	13	2,7	203	3,3	
Informação ignorada	06	0,1	03	0,6	09	0,1	
Anos de estudo							< 0,001
Nenhum	93	1,7	17	3,5	110	1,8	
De 1 a 7 anos	1.729	30,8	159	32,9	1.888	31,0	
8 anos ou mais	2.280	40,7	108	22,4	2.388	39,2	
Informação ignorada	1.507	26,9	199	41,2	1.706	28,0	
Forma/local de diagnóstico							< 0,001
Pronto-socorro ou hospital	3.686	65,7	301	62,3	3.987	65,4	
Serviços ambulatoriais	1.683	30,0	118	24,4	1.801	29,6	
Descoberta após o óbito	140	2,5	26	5,4	166	2,7	
Busca ativa ou investigação de contatos	58	1,0	35	7,2	93	1,5	
Informação ignorada	42	0,7	03	0,6	45	0,7	
Forma clínica ^a^							< 0,001
Pulmonar	3.202	57,1	362	75,1	3.564	58,5	
Extrapulmonar	1.337	23,8	54	11,2	1.391	22,8	
Pulmonar e extrapulmonar ou disseminada	1.070	19,1	66	13,7	1.136	18,7	
Diabetes associada	107	1,9	12	2,5	119	2,0	0,379
Transtorno mental associado	58	1,0	09	1,9	67	1,1	0,094
Alcoolismo associado	539	9,6	142	29,4	681	11,2	< 0,001
Tabagismo associado	146	2,6	28	5,8	174	2,9	< 0,001
Drogadição associada	544	9,7	154	31,9	698	11,5	< 0,001
Encerramento do tratamento ^b^							< 0,001
Cura	3.018	55,5	145	32,7	3.163	53,8	
Óbito	1.212	22,3	111	25,1	1.323	22,5	
Abandono	1.043	19,2	173	39,1	1.216	20,7	
Falência ou resistência	41	0,8	-	0,0	41	0,7	
Transferência para outro estado/país	58	1,1	05	1,1	63	1,1	
Tratamento não encerrado ou ignorado	65	1,2	09	2,0	74	1,3	

^a^ Um caso com informação ignorada sobre a forma clínica não foi incluído (n = 1).

^b^ Casos sem informação sobre o início de tratamento não foram incluídos (n = 212).

Fontes: Sistema de Controle de Pacientes com Tuberculose (TBWeb, 2017); Fundação Sistema Estadual de Análise de Dados (Seade, 2017).

## DISCUSSÃO

A diminuição da incidência da coinfecção TB-HIV no período analisado evidencia um importante avanço no controle da TB e, sobretudo, do HIV, relacionado à introdução oportuna da terapia antirretroviral (TARV) e melhoria do acesso aos serviços de referência ^5 , 19 , 20^ . Contudo, aspectos relacionados ao diagnóstico da TB e ao encerramento do tratamento, com destaque para a grande parcela de diagnósticos realizados em serviços hospitalares e as altas taxas de abandono e óbito, refletem as dificuldades em se efetivar a busca ativa de casos, o diagnóstico precoce e o tratamento efetivo da coinfecção TB-HIV. A análise espacial permitiu identificar áreas com alta incidência, que devem ser prioritárias para intervenções de controle em nível individual e coletivo. Destaca-se que algumas populações são ainda mais afetadas pela coinfecção, como a população sem residência fixa, sendo fundamental a elaboração de estratégias de controle que considerem as singulares vulnerabilidades destes indivíduos, facilitando, assim, o seu acesso à assistência à saúde.

A compreensão sobre a dinâmica da coinfecção TB-HIV demanda a testagem oportuna para o HIV de todos os indivíduos com diagnóstico de TB, conforme é preconizado no Brasil ^21^ . A ampliação da testagem no MSP nos anos mais recentes indica uma melhor organização dos serviços de assistência à TB e ao HIV/aids e o aprimoramento das ações de controle da TB e do HIV/aids ^5 , 15 , 19^ .

O declínio significativo na proporção de casos de TB coinfectados pelo HIV e na taxa de incidência da coinfecção identificado no presente estudo é consistente com os dados de morbidade por TB e HIV/aids no MSP ^15^ . Nas últimas décadas, o Brasil, assim como as Américas e o mundo, apresentou tendência de queda nas taxas de incidência de TB e na mortalidade pelo agravo ^22^ , bem como também decorreu ligeira redução na incidência da TB no período de 2006 a 2013 no MSP ^19^ . Nessa perspectiva, as taxas de incidência de HIV/aids também exibiram decréscimo no MSP nos anos mais recentes ^20 , 23^ , associadas à ampliação do acesso ao diagnóstico precoce da infecção pelo HIV e à TARV ^24 , 25^ .

A presença da coinfecção pelo HIV, cerca de duas vezes maior nos casos de TB sem residência fixa do que na população com moradia fixa, pode ser reflexo do impacto da epidemia de HIV/aids nesse primeiro grupo, cuja prevalência da infecção pelo HIV é de 4,9%, enquanto a população brasileira apresenta uma taxa de 0,6% no mesmo período ^26^ . Somando-se tantas outras vulnerabilidades e condições de exclusão social, a população em situação de rua infectada pelo HIV apresenta um risco extremamente elevado de adoecimento por TB e um precário acesso ao cuidado em saúde ^21 , 27^ .

No sistema de informação TBWeb, principal fonte de dados deste estudo, a entrada do dado de endereço do paciente é padronizada, não sendo permitida a livre digitação, e há como referência uma base de logradouros, condicionando, assim, a entrada das informações e aumentando a precisão da geocodificação dos casos ^28^ . O uso de ferramentas de análise espacial possibilitou a descrição da dispersão do agravo no território e a identificação de aglomerados de casos, que podem ser observados nos mapas das figuras 2 e 3. A dependência espacial segue o princípio de que a maior parte das ocorrências apresentam uma relação que depende da distância entre si, ou seja, de sua distribuição no território ^18^ . O índice de Moran global estatisticamente significativo revelou a existência de autocorrelação espacial positiva na incidência da coinfecção TB-HIV, e o índice de Moran local possibilitou a delimitação dos aglomerados espaciais.

Ao formular medidas de prevenção e controle para a coinfecção TB-HIV, há que se considerar os aspectos determinantes da transmissão e do controle do HIV nos grandes centros urbanos. Alguns estudos descreveram os padrões de distribuição espacial do HIV/aids no MSP ^20 , 23^ , que se assemelham ao observado na coinfecção TB-HIV no presente trabalho. É fundamental que os programas de controle da TB e do HIV/aids configurem ações colaborativas, tanto na ampliação do acesso ao diagnóstico da infecção pelo HIV e introdução oportuna da TARV, quanto na prevenção da TB por meio da investigação e do tratamento da infecção latente, além do enfoque nos grupos mais vulneráveis à infecção e ao adoecimento ^1 , 5 , 21^ .

O padrão geoespacial da TB em São Paulo, que revela áreas fortemente afetadas, como o centro da cidade e áreas periféricas das regiões Norte e Leste ^15 , 19^ , também se aproxima à distribuição espacial das taxas de incidência de coinfecção TB-HIV observadas no presente estudo. Um fator de risco imprescindível à infecção pela TB é o contato com pessoas com a doença ativa, ou seja, indivíduos que moram ou trabalham em ambientes com altas cargas da doença estão sob maior risco de exposição ao bacilo ^16^ . Nesse sentido, conhecer as áreas com alta incidência de TB pode contribuir para a busca ativa dos casos e a quebra da cadeia de transmissão; no caso das populações com maior risco, como os indivíduos que vivem com HIV/aids, isso se torna ainda mais necessário ^7^ .

Em estudo recente realizado em Uganda ^29^ , foram identificados aglomerados espaciais de cada agravo (TB e HIV/aids) e da coinfecção TB-HIV, e concluiu-se que as taxas de TB eram positivamente influenciadas no território pelas taxas de HIV e vice-versa, ou seja, seria necessária a abordagem simultânea de ambos os agravos para seu manejo efetivo. Outra análise realizada no Quênia ^30^ também identificou concentração heterogênea de casos de coinfecção TB-HIV no país e ressaltou a importância de intervenções mais focadas nessas regiões para uma melhor alocação de recursos.

São Paulo é a cidade mais populosa do Brasil ^13^ e possui alta densidade demográfica, com destaque para as áreas mais centrais, que apresentam importante aglomeração urbana, seja de moradores, trabalhadores, unidades de saúde e usuários do transporte público, o que acarreta intenso fluxo e encontro de pessoas, além de maior possibilidade de transmissão de doenças como a TB ^6 , 31^ . Todos os DA que compõem o aglomerado de alto risco para a coinfecção TB-HIV no presente estudo apresentam altas densidades demográficas ^32^ , aspecto que tem sido relacionado às altas taxas de TB no Brasil ^33^ .

Destaca-se ainda que, ao estudar um agravo de saúde com forte e reconhecida determinação social como a TB, a análise espacial pode contribuir para o entendimento da situação de saúde dos indivíduos, visto que o território ultrapassa o significado de um espaço puramente geográfico, refletindo também a sua inserção na sociedade e os seus potenciais de enfrentamento no processo saúde-doença ^9 , 18^ . Entretanto, em estudos brasileiros ^34 , 35^ foram identificadas altas taxas de coinfecção TB-HIV também em regiões com bons indicadores socioeconômicos, o que difere do panorama tradicional encontrado na TB, que impacta, sobretudo, as populações com forte exclusão social. Um indicador comumente utilizado para avaliar o nível de desenvolvimento de países ou regiões é o índice de desenvolvimento humano. Esse índice, que varia entre 0 e 1, quando utilizado em escala municipal é denominado índice de desenvolvimento humano municipal (IDHM), composto por três dimensões: longevidade, educação e renda ^36^ . Ao analisar as regiões do aglomerado espacial de alto risco para a coinfecção TB-HIV, nota-se que apresentam alto desenvolvimento humano, e inclusive desenvolvimento muito alto quando avaliadas somente as dimensões de renda e longevidade ^36^ . Isto pode indicar um contexto social diferenciado nas regiões mais impactadas pela coinfecção TB-HIV, padrão destoante daquele encontrado na literatura em relação à TB ^33^ . Compreender os aspectos que determinam a concentração dos casos de coinfecção TB-HIV no MSP pode contribuir para o controle da TB nas regiões com maior risco e facilitar a formulação de políticas de saúde para uma organização mais efetiva dos serviços de assistência à TB e ao HIV/aids.

O perfil sociodemográfico da população coinfectada evidencia a prevalência do sexo masculino e da faixa etária economicamente ativa ^7 , 37^ . No presente estudo, a população sem residência fixa apresentou proporção de pretos ou pardos duas vezes maior que a de brancos, corroborando com outros estudos sobre TB e HIV/aids em indivíduos em situação de rua ^19 , 26 , 38^ . Entende-se que as desigualdades raciais são determinantes da iniquidade em saúde, pois impactam de forma contundente nas relações sociais, na autoestima e no acesso ao cuidado e à assistência. A baixa escolaridade da população em situação de rua e de parcela importante dos indivíduos com moradia fixa já foi descrita em estudos sobre a coinfecção TB-HIV e pode repercutir no entendimento dos aspectos relacionados à doença e ao tratamento ^7 , 38^ .

O diagnóstico realizado em serviços de urgência e emergência na maioria dos casos pode refletir estágios avançados da doença, quando, por fim, o diagnóstico é feito e o tratamento é iniciado. De forma frequente, a infecção pelo HIV é diagnosticada concomitantemente à TB, implicando um enorme impacto na vida desses indivíduos, que, além de lidar com dois agravos infecciosos debilitantes, cada um com a sua complexidade e tratamento específicos, já apresentam menores chances de cura do que os indivíduos não infectados pelo HIV ^39^ . A parcela diagnosticada após o óbito, em especial entre os indivíduos sem residência fixa, evidencia a grande dificuldade de acesso e vínculo ao serviço de saúde vivenciada por essa população ^26 , 27^ , que morre sem o diagnóstico e a oportunidade de tratar a doença.

Observa-se que a predominância da forma pulmonar, presente em quase 90% da população coinfectada sem residência fixa, relaciona-se ao maior risco de transmissão, que é ampliado por aspectos como a exposição ambiental, condições precárias de acomodação e alimentação e situações de aglomeração ^27^ . Apesar disso, a forma extrapulmonar também apresentou relevante magnitude. De fato, em indivíduos com comprometimento imunológico importante e aids avançada, as formas extrapulmonares da TB são mais comuns ^7 , 37 , 40^ . Todavia, o uso da TARV de forma adequada possibilita a manutenção da imunocompetência e está associada à diminuição da incidência de TB ^40^ .

No Brasil, o cuidado com as pessoas que vivem com HIV/aids possui caráter descentralizado, sendo idealmente conduzido na atenção básica e nos serviços de assistência especializada (SAE), desde o diagnóstico da infecção, introdução e monitoramento da TARV até a prevenção e o tratamento dos agravos associados ^5 , 21^ . Nesse sentido, a investigação da TB em todos os atendimentos ao indivíduo com HIV/aids é uma ação primordial para o diagnóstico oportuno dos doentes com TB ativa, sendo útil também na prevenção do adoecimento por TB, pois possibilita o diagnóstico e o tratamento da infecção latente ^41^ .

O alcoolismo e a drogadição foram observados em parcela importante da população, sobretudo entre os indivíduos em situação de rua. Um estudo realizado em Lima, no Peru ^37^ , apontou maior frequência de uso destas substâncias nos indivíduos coinfectados do que nos casos de TB HIV-negativos. A literatura também sugere que o uso de substâncias psicoativas está relacionado ao desenvolvimento e à transmissão da TB, ao passo que diminui a defesa imunológica dos indivíduos que fazem uso delas ^16^ . Além disso, é notável a associação entre o uso nocivo de álcool e outras drogas e os desfechos desfavoráveis de tratamento, como o abandono ^16 , 27 , 38^ . Destaca-se a importância da investigação desses agravos no diagnóstico e durante o tratamento, como forma de identificar as dificuldades para a adesão e fortalecer o vínculo com a equipe de saúde.

No presente estudo, um total de 97,7% dos casos possuía o desfecho de tratamento registrado na FN-TB e, nesse grupo, foram identificadas taxas de cura muito abaixo do preconizado (≥ 85%) e inferiores às dos indivíduos não infectados pelo HIV no mesmo contexto social ^19^ . Na associação entre a TB e o HIV, o risco de morte durante o tratamento é 3 a 19 vezes maior do que em indivíduos não infectados pelo HIV ^39^ . As menores proporções de cura nos coinfectados também são corroboradas por outros estudos ^37 , 38 , 40 , 42^ , e nessa perspectiva são necessárias políticas de saúde mais arrojadas, com foco na população que vive com HIV e sofre com TB, com estratégias integradas e que contribuam para o diagnóstico precoce, o manejo adequado dos agravos e a adesão ao tratamento.

Nos indivíduos coinfectados pelo HIV, o tratamento diretamente observado (TDO) tem se mostrado ainda mais necessário, em razão da complexidade dos agravos e da possibilidade de interações medicamentosas com a TARV ^2 , 21^ , e deve ser oferecido a todos os pacientes com TB ^21^ . Em 2017, entretanto, dentre os casos coinfectados que faziam uso de TARV, somente 22,1% estavam em TDO e, no grupo que não utilizava TARV, a proporção que tratava a TB de forma supervisionada era ainda menor (13,9%) ^5^ . A vigilância epidemiológica possui um papel fundamental na articulação com a rede assistencial e no monitoramento dos casos, podendo contribuir para intervenções oportunas da equipe de saúde e para a expansão do TDO, fortalecendo o controle da doença no território ^5^ .

Na população sem residência fixa, notam-se desfechos de tratamento ainda mais desfavoráveis, com quase o dobro da taxa de abandono da população com moradia fixa e a cura atingida por somente um terço dos indivíduos em tratamento, achados corroborados por outros trabalhos ^27 , 38^ . Considerando as inúmeras vulnerabilidades sociais, individuais e programáticas vivenciadas por esses indivíduos, podem-se destacar algumas, como a precária alimentação diária, o abuso de álcool e outras substâncias psicoativas, as dificuldades de acesso e vínculo ao serviço de saúde e o estigma e preconceito perante a sociedade ^26 , 27 , 38^ , que podem dificultar o acesso ao cuidado em saúde e contribuir para a baixa adesão ao tratamento.

Nesse sentido, estratégias diferenciadas são essenciais para lidar com o desafio da adesão pela população em situação de rua. A articulação intersetorial, com parcerias com a assistência social, organizações sociais e o apoio da própria sociedade civil, é determinante para o sucesso das ações ^15^ . Destaca-se a relevância do TDO para potencializar a adesão por meio da aproximação com a equipe de saúde e do oferecimento de incentivos sociais, tão importantes para esses indivíduos que vivenciam um contexto social singular e desafiador para o tratamento e controle da doença ^27 , 38 , 43^ . A população em situação de rua deve ser considerada na formulação de políticas específicas de controle da coinfecção TB-HIV, sendo também imprescindível que os profissionais e os serviços de saúde estejam preparados para atender às suas demandas, não somente atentando para os aspectos clínicos, mas também integrando a abordagem social e o fortalecimento da cidadania.

É fundamental ressaltar que os estudos ecológicos apresentam algumas limitações, pois com a análise dos dados de forma agregada não é possível afirmar que as conclusões ocorrem de forma semelhante no nível individual ^44^ . O DA como unidade espacial de análise apresenta grande extensão territorial e com heterogeneidade de características; todavia, por ser uma divisão administrativa, o seu uso pode facilitar a tomada de decisões durante o planejamento e a organização das ações de controle com enfoque nas áreas prioritárias.

Além disso, os dados utilizados são secundários, provenientes de um sistema de informação de vigilância epidemiológica alimentado pelas notificações dos casos, e podem conter falhas de preenchimento, dados desatualizados e falta de informações; portanto, as análises devem ser interpretadas com cautela. A precariedade da completude das informações pode dificultar o real entendimento de quem está adoecendo e, assim, interferir no planejamento das ações de controle ^45^ , o que pode ser modificado e aprimorado por meio da educação, seja durante a formação acadêmica ou na capacitação continuada dos profissionais, e também pela qualificação das informações por meio de relacionamentos probabilísticos entre as bases de dados utilizadas na vigilância epidemiológica ^5^ .

Ressalta-se que o conhecimento do uso ou não da TARV poderia proporcionar mais elementos para o entendimento da evolução clínica desses indivíduos, porém essa variável somente foi incluída na FN-TB em 2016, impossibilitando a análise nos indivíduos incluídos neste estudo; entretanto, ela poderá agregar aspectos importantes em análises futuras sobre o agravo ^5^ . Por fim, acredita-se que os resultados deste estudo podem auxiliar na organização dos serviços de assistência à saúde e no aperfeiçoamento das atividades colaborativas pelos programas de controle de TB e de HIV/aids, fortalecendo as ações de prevenção, diagnóstico e tratamento da coinfecção TB-HIV.
